# Diagnostic Performance of Somatostatin Receptor-directed PET/CT for Tumor-induced Osteomalacia

**DOI:** 10.1007/s11307-026-02101-z

**Published:** 2026-05-04

**Authors:** Marieke Heinrich, Aleksander Kosmala, Alexander Dierks, Franca Genest, Elena Gerhard-Hartmann, Lukas Haug, Silke Achtziger, Peter Raab, Andreas K. Buck, Constantin Lapa, Kerstin Michalski, Lothar Seefried

**Affiliations:** 1 Department of Nuclear Medicine, University Hospital Wuerzburg, Wuerzburg, 97080 Germany; 2 Department of Nuclear Medicine, Faculty of Medicine, University of Augsburg, University Hospital Augsburg, Augsburg, Germany; 3 Experimental and Clinical Osteology, Orthopedic Department, Orthopedic Center for Musculoskeletal Research, Julius-Maximilians-University of Wuerzburg, Wuerzburg, Germany; 4 Institute of Pathology, Julius-Maximilians-University of Wuerzburg, Wuerzburg, Germany; 5 Dr. Senckenberg Institute of Pathology, University Hospital Frankfurt of the Goethe University, Frankfurt, Germany

**Keywords:** Oncogenic osteomalacia, Hypophosphatemia, Renal Phosphate wasting, FGF-23, Total lesion uptake

## Abstract

**Aim:**

To evaluate the efficacy of somatostatin receptor (SSTR)-directed PET/CT in localizing phosphaturic mesenchymal tumors (PMT) in patients with suspected tumor-induced osteomalacia (TIO) and to explore relationships between imaging parameters and biochemical markers.

**Methods:**

This retrospective analysis included 20 patients with suspected TIO, undergoing SSTR-directed PET/CT. Imaging findings and laboratory markers were assessed. SSTR-positive tumors were resected, while patients without detectable tumor, but persistent renal phosphate wasting, continued on medical treatment. Follow-up assessments included laboratory values and clinical examinations.

**Results:**

PMT were detected on PET in 12 patients (60%), were resected, and confirmed immunohistochemically. Phosphorus levels (*r* = 0.26; *p* = 0.03), tubular reabsorption of phosphate (TRP; *r* = 0.77; *p* < 0.01) and tubular maximum of phosphate reabsorption over GFR (TmP/GFR; *r* = 0.44; *p* = 0.07) were lower in patients with detected PMT compared to those without. Elevation of fibroblast growth factor 23 (FGF23) was not significantly higher in PMT positive vs negative patients (253% vs 134% above the upper limit of normal; *p* = 0.97). Total lesion uptake (TLU) negatively correlated with TmP/GFR (*r* = -0.71; *p* = 0.03). A maximum standardized uptake value (SUVmax) threshold of 7.6 differentiated PMT from bone fractures (83% sensitivity; 100% specificity). Post-resection follow-up confirmed clinical cure in all cases.

**Conclusion:**

In SSTR-directed PET/CT, a distinction between PMT and bone fracture may be possible with a SUVmax threshold of 7.6. The integration of PET derived TLU, along with TmP/GFR may improve diagnosis and treatment planning for TIO.

**Supplementary Information:**

The online version contains supplementary material available at 10.1007/s11307-026-02101-z.

## Introduction

Tumor-induced osteomalacia (TIO) is a rare paraneoplastic syndrome characterized by the overproduction of fibroblast growth factor 23 (FGF23), typically by small phosphaturic mesenchymal tumors (PMT). This condition leads to renal phosphate wasting, hypophosphatemia, and impaired bone metabolism, resulting in osteomalacia [1, 2]. Patients with TIO often present with progressive musculoskeletal pain, fatigue and multiple fractures limiting activities of daily live [1–3]. The nonspecific nature of these symptoms, combined with the rarity of the condition, frequently leads to misdiagnosis and significant delays in proper treatment. On average, patients experience a delay of three to six years between symptom onset and accurate diagnosis [4–6]. Diagnosis of TIO requires a combination of clinical suspicion, biochemical evaluation, and imaging studies. Key laboratory findings include hypophosphatemia, increased or inappropriately FGF23 levels in the reference level and decreased tubular reabsorption of phosphate (TRP) along with decreases in tubular maximum phosphate reabsorption capacity (TmP/GFR) [2, 7]. Localization of the causative tumor is crucial for management but often challenging due to the small size, slow growth and variable location of PMT [8]. Anatomical imaging including CT and MRT techniques are insufficient to identify these functionally active neoplasms. The discovery that PMT typically express high levels of somatostatin receptors has revolutionized the diagnostic approach to TIO [9–11]. In recent years, somatostatin receptor (SSTR)-directed PET/CT has emerged as a promising tool for tumor localization in TIO, including DOTA[^68^Ga]-[Tyr^3^]Octreotat (^68^Ga-DOTATATE) and DOTA(0) [^68^Ga]-Phe(1)-Tyr(3)) octreotid ([^68^Ga]Ga-DOTATOC) [9, 12–14]. Treatment options for TIO primarily involve surgical resection of the tumor, which can lead to rapid normalization of phosphate levels and clinical improvement [2]. In cases where the tumor cannot be localized or completely resected, medical management traditionally involved phosphate supplementation and active vitamin D analogs, but is currently conducted using the recently approved anti-FGF23 monoclonal antibody burosumab [7, 8]. As research in this field continues to evolve, further refinements in interpretation of findings in SSTR-directed PET/CT and further understanding of PET findings in combination with biochemical evaluations are warranted.

## Materials and Methods

### Study Setting

This retrospective analysis included patients with suspected TIO cared for at the Osteology Department of the Musculoskeletal Center in Wuerzburg, Germany between July 2012 and August 2024. All patients were examined using SSTR—directed PET/CT with [^68^Ga]Ga-DOTATOC at the University Hospitals of Wuerzburg Germany. Patients with evidence of PMT in SSTR-directed PET/CT underwent surgical resection, patients without evidence of PMT but persistent renal phosphate wasting were continued or started on phosphate/vitamin D supplementation therapy or burosumab. The local Ethics Committee of Julius-Maximilians University, Wuerzburg, Germany waived the need for further approval due to the retrospective character of the study (waiver no. 20241002 01).

### Patients

In all patients, genetic causes for phosphate wasting were excluded. Standard clinical assessment before referral to SSTR-directed PET/CT included medical history, clinical examination, bone density evaluation using Dual X-ray Absorptiometry (DXA) and morning fasting laboratory tests comprising relevant parameters for bone and mineral metabolism, i.e. calcium, phosphate, creatinine, alkaline phosphatase (ALK), parathyroid hormone (PTH), N-terminal telopeptide (NTx), procollagen type I N propeptide (PINP), 25OH vitamin D and 1,25 OH vitamin D3 and urinary analysis for phosphate, calcium and creatinine to calculate TRP, TmP/GFR and Calcium/Creatinine ratio. Assessment of FGF23 utilized different assays over time, typically c-terminal FGF23 (Human FGF-23 (c-Term) ELISA Kit, 2nd Generation Enzyme-Linked ImmunoSorbent Assay; Immutopics Inc, San Clemente, CA 92673, USA) until 2020 and both c-term (FGF23 (c-terminal) multi-matrix ELISA, Biomedica, Vienna, Austria) and intact FGF23 (FGF23 (intact) human ELISA; Biomedica, Vienna, Austria) as of 2020. To compare these values, we used the relative deviation from the upper limit. The maximum interval between the determination of blood values and SSTR-directed PET/CT was set at 30 days. In three patients we had missing values for NTx and TRP and in two patients we had missing values for TmP/GFR, see Supplemental Table 1 for all information.

### Immunohistochemistry

Histological sections (2 μm) of formalin-fixed paraffin-embedded (FFPE) tissue were cut and stained with haematoxylin and eosin (HE) for routine histological evaluation. SSTR2A immunohistochemistry (IHC) was performed on FFPE tissue slides using a polyclonal antibody (RBK 046–05, Zytomed, Berlin, Germany; dilution 1:20) and an automated immunostainer (BOND-III, Leica Biosystems, Wetzlar, Germany) according to the manufacturers' instructions and standard protocols. Nuclear contrast was achieved by haematoxylin counterstaining. Immunohistochemistry was evaluated using an Olympus BX53 microscope. Membranous staining was considered positive, and the proportion and staining intensity of positive tumor cells was evaluated, using the previously described modified immunoreactive score of Remmele and Stanger (IRS) method [15]. The proportion of stained cells was categorized as follows: 0 for 0%, 1 for 1–9%, 2 for 10–50%, 3 for 51–80% and 4 for more than 80%. The intensity of staining was categorized as follows: 1 for weak intensity, 2 for medium intensity and 3 for strong intensity. The final score was calculated as the product of intensity and proportion and has a range of 0–12 [16, 17].

### Imaging Procedure and Analysis

Imaging was performed using dedicated PET/CT systems including the Biograph mCT 128 (in *n* = 11 patients) or Biograph mCT 64 (*n* = 9; all Siemens Medical Solution, Erlangen, Germany). The patient cohort consisted of 20 individuals receiving [^68^Ga]Ga-DOTATOC. The administered activity was 133 ± 13 MBq intravenously, with a 45–60 min uptake period. CT was performed with (*n* = 13) or without (*n* = 7) iodine-containing contrast, utilizing automatic tube current modulation. The reference mAs was 35 for low-dose (*n* = 7) and 160 for full-dose (*n* = 13) scans, respectively. Tube voltage was set at 120 keV for the mCT 64 and at 100 keV for the mCT 128. The pitch values were 1.4 for the mCT 64, 0.8 for the mCT 128. The rotation time was 0.5 s, with a reconstructed axial slice thickness ranging from 3.0 to 5.0 mm across all systems. The scan coverage ranged from the top of the skull to the tips of the toes, with the arms positioned next to the body. PET emission data were acquired in three-dimensional mode with a 200 × 200 matrix, with an emission time of 2–5 min per bed position. After decay and scatter correction, the data were reconstructed iteratively with attenuation correction using TrueX (HD-PET; Software VG62E) for the mCT64 and TrueX + TOF (UltraHD PET; Software VG80C) for the mCT128 implemented by Siemens Healthineers, Erlangen, Germany. The PET/CT section thickness was consistently 5 mm. PET-based quantification was also conducted by manually segmenting sites of disease using a dedicated software package (syngo.via, Siemens Healthineers, Erlangen, Germany), providing averaged peak, maximum and mean standardized uptake value (SUVpeak/SUVmax/SUVmean). Tumor volume (TV) was calculated by drawing a spherical volume of interest, with an automatically adaption for a three-dimensional VOI at a 40% isocontour [18], representing the molecular tumor volume rather than an anatomical volume. Total lesion uptake (TLU) was calculated with TV and SUVmax (TV*SUVmax).

### Statistical Analysis

We used GraphPad Prism version 10.4.0 (GraphPad Software, San Diego, California, United States) for statistical analyses. Unless otherwise described data are presented as median and range or with their 95% confidence interval (CI) in parentheses. Statistical analysis of relationships between laboratory values and PET-based values was performed using the Spearman rank test. Differences in paired samples were assessed with the Wilcoxon matched-pairs signed rank test, while differences in unpaired samples were analyzed using the Mann–Whitney U test. The diagnostic accuracy of PET-based values for PMT was determined using receiver operating characteristics (ROC) curves, and the optimal threshold was identified using the Youden Index J [19].

## Results

### Patient Characteristics

The patient cohort included 20 patients (10 males, 10 females, median age 61 years ± 12 years) with suspected TIO. All patients showed typical clinical symptoms and laboratory findings for TIO. Baseline characteristics of the patients are outlined in Table 1. In fifteen patients, PMT-derived laboratory values were determined on the day of imaging and in the remaining six patients with a median interval of 21 days (range, 1–29 days). For PET-based values and laboratory values see Supplemental Table 2, for patient-based values see Supplemental Table 1.

**Table 1:** Patient characteristics

		median	range
Age at diagnosis [years]		61	20—75
Height [cm]		171	139—192
Weight [kg]		72	40—105
BMI [kg/m^2^]		27	21—33
Time between first symptoms and diagnosis [years]		5	1—20
		number [*n*]	percentage [%]
Exclusion of a genetically determined phosphate wasting		20	100
Supplementation therapy at PET/CT Scan	Phosphate salts	15	75
	Cholecalciferol	16	80
	Calcidiol	1	5
	Calcitriol	15	75
Surgical intervention prior to PET/CT		7	35
	incomplete resection	1	5
	Biopsy	3	15
	Biopsy and resection	1	5
	Biopsy and two resections	1	5
	Biopsy and three resections	1	5

*BMI* body-mass-index

Seven out of 20 patients (35%) had previously undergone surgical intervention for TIO in other centers and 16/20 were receiving supplementation therapy consisting of active vitamin D (*n* = 16) and phosphate salts (*n* = 15) at the time of imaging. In twelve patients, PMT was detected in SSTR-directed PET/CT. Out of eight patients without detection of PMT in SSTR-directed PET/CT, one patient was eventually diagnosed with FGF23-independent phosphate wasting with stable laboratory values under conventional inactive vitamin D (cholecalciferol) supplementation. In the remaining seven patients, the diagnosis of TIO was confirmed. Four patients were started on burosumab therapy and showed clinical improvement, further supporting the diagnosis of TIO. In two patients with partial improvement under supplementation of phosphate, calcitriol and cholecalciferol, transition to burosumab therapy is planned. One patient, who was diagnosed with suspected TIO due to recurrent typical laboratory findings but without existing symptoms, is currently untreated. The median follow up period was 19 months (range, 3–92 months). No patients experienced recurrence of signs and symptoms during follow up.

### PMT in SSTR-directed PET/CT

In twelve out of 20 patients (60%) PMT was detected by SSTR-directed PET/CT and the diagnosis was confirmed in all twelve patients through resection and histological analysis including immunohistochemistry for SSTR2A. Most PMT were located at the lower limb (*n* = 6; Supplemental Table 3) and in the soft tissue (*n* = 8). In this cohort, no PMT was located at the head and neck or the upper limb. For all results see Supplemental Table 3.

### Histological Expression of SSTR2A Receptors

**Fig. 1:**
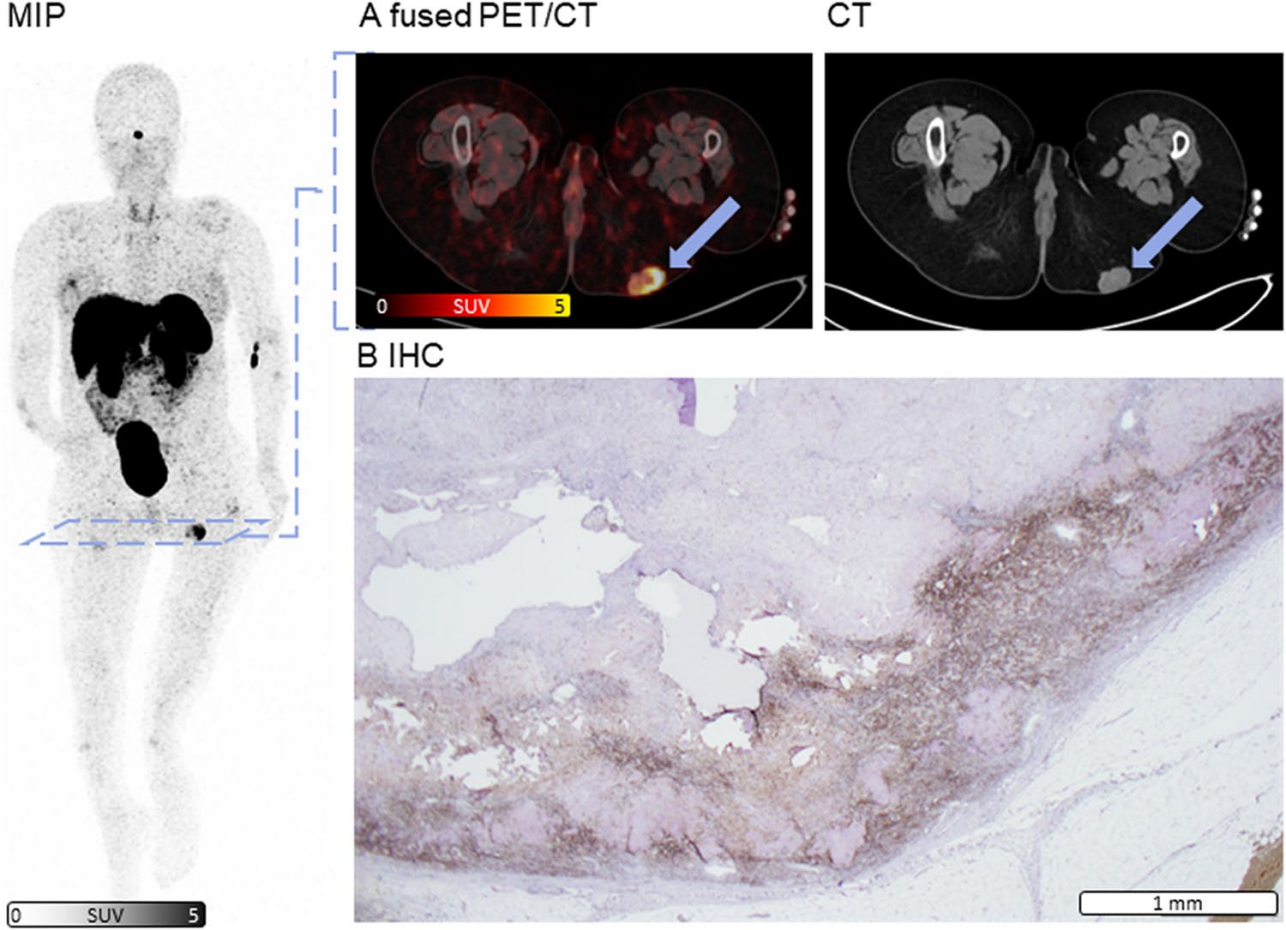
Example for distribution of [^68^Ga]Ga-DOTATOC in SSTR-directed PET/CT (fused PET/CT and CT; **A**) and corresponding SSTR2A IHC (**B**) in patient number 8. *DOTATOC* DOTA(0)-Phe(1)-Tyr(3))octreotid, *IHC* immunohistochemistry, *MIP *maximum intensity projection, *SSTR* somatostatin receptor, *SUV* standardized uptake value

Immunohistochemically, all evaluable tumors (11/11) showed positive staining for SSTR2A at different levels. In one patient, SSTR2A IHC could not be evaluated validly due to fixation artefacts. Median IRS score was 6 (range 1–12), see Supplemental Table 4 for detailed information. Predominantly larger tumors showed an inhomogeneity of SSTR expression in PET/CT probably due to matrix deposits, which was also reflected in the distribution of the immune reaction as shown by IHC (exemplified in Fig. 1 as an example). The IRS showed a fair to good correlation with SUVmax (*r* = 0.51; *p* = 0.11), SUVpeak (*r* = 0.34; *p* = 0.31) and SUVmean (*r* = 0.52; *p* = 0.10) as well as the MTV (*r* = −0.49; *p* = 0.13). In contrast, no correlation with TLU (*r* = 0.17; *p* = 0.78) was observed.

### PMT of Bone vs Soft Tissue Origin

The median SUVmax of PMT (*n* = 12) was 15.3 (95% CI 7.82–60.30), SUVpeak was 8.12 (95% CI 4.42–20.34), SUVmean was 8.60 (95% CI 4.82–38.35), TV was 1.58 ml (95% CI 1.20- 2.04 ml) and TLU was 10.90 ml (95% CI 7.49–45.90). We analyzed differences in PET-derived parameters between PMT of bone and soft tissue origin and found no significant differences in SUVmax (bone, 21.51 vs soft tissue, 15.30; U = 15.00; *p* = 0.93; Supplemental Fig. 1a), SUVpeak (12.29 vs 7.28; U = 15.00; *p* = 0.93; Supplemental Fig. 1b), TV (2.10 ml vs 1.39 ml; U = 10.50; *p* = 0.39; Supplemental Fig. 1c) and TLU (11.21 ml vs 10.90 ml; U = 15.00; *p* = 0.93; Supplemental Fig. 1d).

### PET-based Parameters and Biochemical Evaluations

**Fig. 2:**
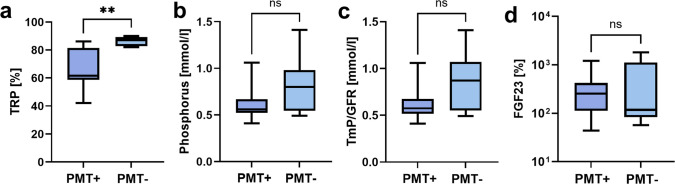
Differences between patients with (PMT +) and without PMT (PMT-) in SSTR-directed PET in TRP (**A**; *p* < 0.01), phosphorus (**B**), TmP/GFR (**C**) and FGF23 (**D**). *FGF23* fibroblast growth factor 23, *PMT* phosphaturic mesenchymal tumors, *SSTR* somatostatin receptor, *TRP* tubular reabsorption of phosphate, *TmP/GFR* tubular maximum of phosphate reabsorption capacity

For exploratory purposes, association between PET-derived parameters and laboratory parameters were investigated. In twelve patients, a benign PMT was detected in SSTR-directed PET/CT (PMT +) and in eight patients no PMT could be located (PMT-). A significant difference in TRP levels was observed between the PMT + group (median 62%) and the PMT- group (median 87%; U = 4; *p* < 0.01; Fig. 2a) with a strong effect (*r* = 0.71). Additionally, there was an insignificant difference in phosphate levels (PMT + median 0.56 mmol/l; PMT- median 0.80 mmol/l; U = 27.50; *p* = 0.06; Fig. 2b) with a medium effect (*r* = 0.26) and an insignificant difference in TmP/GFR with a strong effect (PMT + median 0.58 mmol/l; PMT- median 0.87 mmol/l; U = 16.50; *p* = 0.07; *r* = 0.44; Fig. 2c). There was no significant difference between PMT + and PMT- regarding calcium (2.35 mmol/l vs 2.35 mmol/l; U = 53; *p* = 0.96), FGF23 (253% vs 134%; U = 47; *p* = 0.97; Fig. 2d) and NTx (21.8 BCE/l vs 14.3 BCE/l; U = 20; *p* = 0.32) levels.

**Fig. 3:**
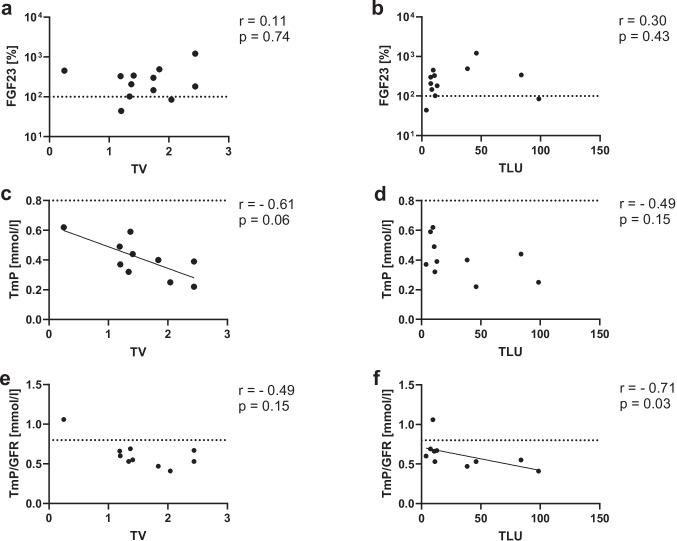
Correlation of FGF23 (**A-B**), TmP (**C-D**) and TmP/GFR **(E-F**) with PET derived parameters TV (**A**, **C**, **E**) and TLU (**B**, **D**, **F**). *FGF23* fibroblast growth factor 23, *SUV* standardized uptake value, *TLU* total lesion uptake, *TmP* tubular maximum of phosphate reabsorption, *TmP/GFR* tubular maximum of phosphate reabsorption capacity, *TV* tumor volume

The PET-derived TLU showed a significant negative correlation with TmP/GFR (*r* = −0.71; *p* = 0.03; *n* = 10; Fig. 3f). We found no significant correlation between TLU and the serum values for calcium, phosphate, FGF23, NTx, TRP and TmP (Fig. 3b, d and Supplemental Table 5). There was no significant correlation between TV and the serum values mentioned above (Fig. 3a, c, e and Supplemental Table 5) and no significant correlation between SUVmax, SUVpeak, SUVmean and the serum values mentioned above, respectively (Supplemental Fig. 2 and Supplemental Table 5).

A TRP less than or equal to 62% was only seen in PMT + patients (*n* = 6). Similarly, fasting phosphorus levels and TmP/GFR of less than or equal to 0.47 mmol/l was only observed in PMT + patients (*n* = 2), respectively.

### PMT vs Fracture

**Fig. 4:**
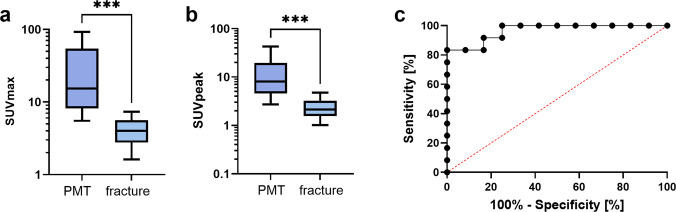
Significant differences in PET-derived parameters SUVmax (**A**; 15.30 vs 4.00; *p* < 0.01) and SUVpeak (**B**; 8.12 vs 2.14; *p* < 0.01) between PMT and bone fracture with ROC curve for a cut off to distinct PMT from fracture (threshold SUVmax 7.6; 83% sensitivity (95% CI 55,20% – 97,04%), 100% specificity (95% CI 75,75% – 100,00%)). *CI* confidence interval, *PET* positron emission tomography, *PMT* phosphaturic mesenchymal tumors, *ROC* receiver operating characteristics, *SUV* standardized uptake value

**Fig. 5:**
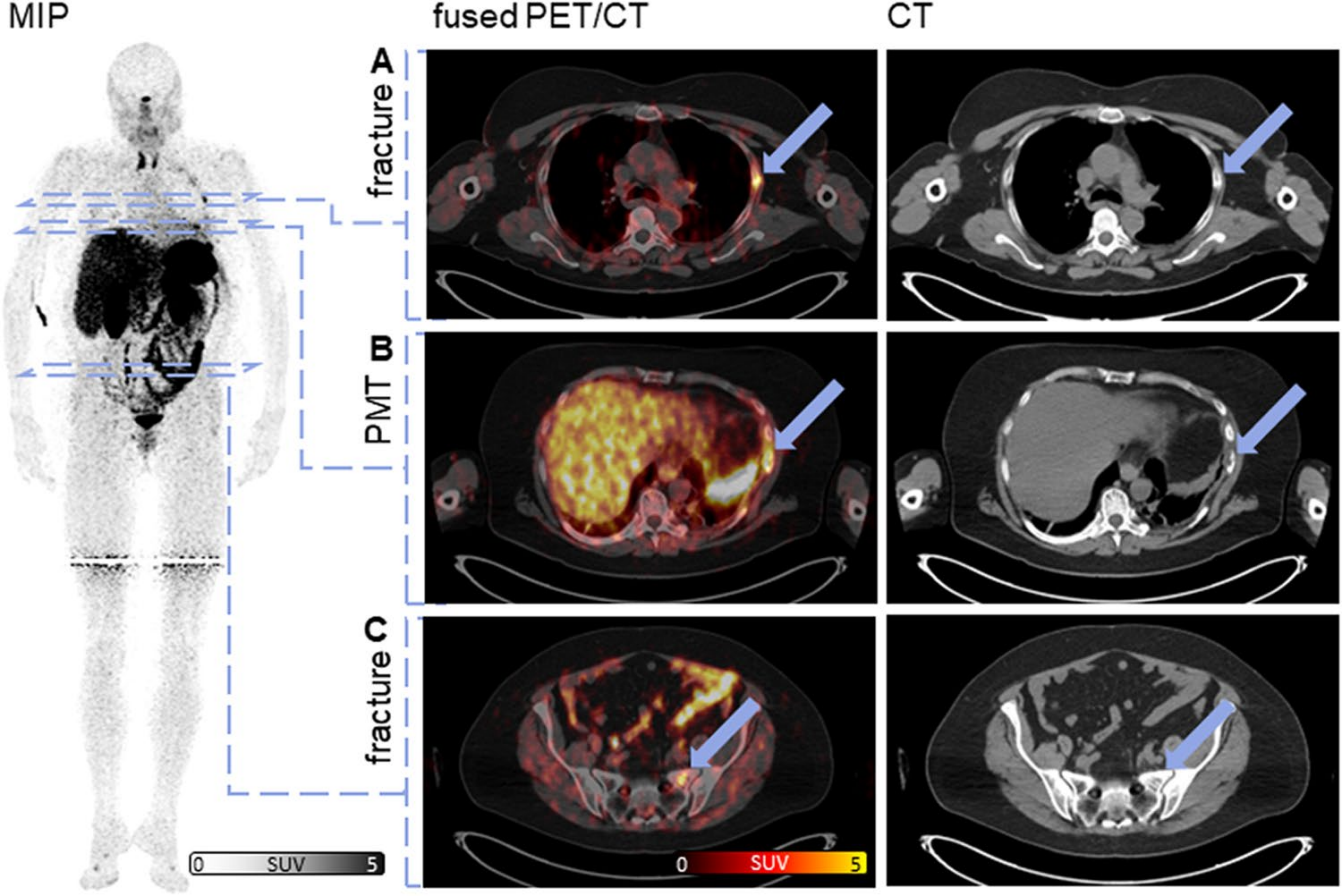
Example of a 63 year old patient (number 11) with osseous PMT (**B**: SUVmax 9.61) and bone fractures (**A**: SUVmax 4.32 and **C**: SUVmax 4.56), depicted in MIP, fused PET/CT and CT. *CT* computer tomography, *MIP* maximum intensity projection, *PET* positron emission tomography, *PMT* phosphaturic mesenchymal tumors, *SUV* standardized uptake value

All twelve patients with detection of PMT in SSTR-directed PET/CT showed bone fractures. Median SUVmax of all bone fractures was 4.00 (95% CI 2.73–5.76) and SUVpeak was 2.14 (95% CI 1.54–3.34). In the analysis of differences in PET-derived parameters between PMT and bone fractures, SUVmax (Fig. 4a) and SUVpeak (Fig. 4b) were significantly higher in PMT than in fractures (SUVmax: PMT 15.30 vs fracture 4.00; Z = −3.06; *p* < 0.01 and SUVpeak PMT 8.12 vs fracture 2.14; Z = −3.06; *p* < 0.01; Fig. 5). To determine a threshold of SUVmax for distinguishing between PMT and fracture, a ROC curve was created (Area under the curve 0.97; 95% CI 0.90–1.00; *p* < 0.01; see Fig. 4c). A SUVmax threshold of 7.6 for the differentiation between PMT and fracture showed a sensitivity of 83% (95% CI 55,20% – 97,04%) and specificity of 100% (95% CI 75,75% – 100,00%).

### Follow Up

In all twelve patients with PMT detection in SSTR—directed PET/CT, the PMT was resected and histologically confirmed afterwards. All patients showed significant clinical improvement after surgery with reduction of pain and improvement in mobility. Compared to the baseline values before resection, patients showed significantly higher fasting phosphorus levels one month after resection (0.66 mmol/l vs 1.32 mmol/l; *n* = 13; *p* < 0.01), higher TRP levels (68% vs 92%; *n* = 8; *p* = 0.02) and higher TmP/GFR (0.57 mmol/l vs 1.32 mmol/l; *n* = 7; *p* = 0.02) respectively.

## Discussion

This study underscores the value of SSTR-directed PET/CT with [^68^Ga]Ga-DOTATOC as a crucial diagnostic tool in localizing PMT in patients with suspected TIO and offers new insights into the relationships between imaging and biochemical parameters.

### PMT in SSTR-directed PET/CT

The results demonstrate a detection rate of PMT using SSTR-directed PET/CT with [^68^Ga]Ga-DOTATOC of 60%, which is consistent with previous studies. El–Maouche et al. showed a detection rate of 55% for [^68^Ga]Ga-DOTATATE [9], Kato et al. 57% for [^68^Ga]Ga-DOTATOC [12] and Paquet et al. 60% for [^68^Ga]Ga-DOTATOC, respectively. In eight patients of our cohort, no PMT was detected. One patient eventually turned out to have FGF23-independent phosphate wasting. Considering only patients with confirmed diagnosis, SSTR-directed PET/CT shows a detection rate of 63%.

In one case, no immunoreactivity was observed in SSTR2A IHC due to suboptimal tissue fixation, which may be partly caused by the peculiar matrix produced by these tumors. Consequently, the result is interpreted as false negative. All other tumors showed SSTR2A expression at different levels in IHC, supporting the findings outlined by Houang et al., analyzing the expression of SSTR2A in 15 PMTs with positive staining in all tumors [11]. However, it cannot be ruled out that fixation artifacts may have affected the accuracy of SSTR2A staining and the resulting immunoreactive score (IRS) in other PMTs in this series as well, albeit to a much lesser extent.

We couldn’t find previous data analyzing the relation between PET-derived TV and TLU and SSTR2A expression in PMT and to the best of our knowledge, no IRS was calculated for PMT before. We found a fair to good correlation of SSTR2A IRS with SUVmax and SUVmean. Consistently, Boy et al. analyzed the relation of SUVmax in [^68^Ga]Ga-DOTATOC-PET/CT with the mRNA expression of the SSTR subtypes in human tissues and found a correlation especially for SUVmax and SSTR2 [20]. Our assessment revealed a negative correlation of SSTR2A IRS and TV. This is consistent with results of Kim et al., showing a negative correlation between histological tumor size and SSTR2 expression in rectal neuroendocrine tumors [21].

While PMT express several markers by IHC, none of these shows a high sensitivity and specificity [22]. The negative correlation between SSTR2A and TV may show the growing impact of other characteristics of PMT with growing size, including other SSTR subtypes, which is also reflected in the wide range of the IRS. However, this hypothesis needs further investigation.

Furthermore, we found no significant differences in SUVmax and SUVpeak between PMTs of soft tissue or bone origin. Again, we couldn`t find any other study which had evaluated these differences, so far.

### PET-based Parameters and Biochemical Evaluations

To the best of our knowledge, so far no study evaluated the association of TRP and TmP/GFR with the PET parameter TLU. We found a significant negative correlation between PET derived TLU and TmP/GFR, potentially indicating an association between tumor size and/or receptor density and renal phosphate wasting. In that regard, one might hypothesize that TLU reflects the endocrinological activity of the tumor tissue or the amount of active tumor cells in a way that higher TLU values correlate with more pronounced impairment of TmP/GFR. The positive correlation between IRS and PET intensity confirms that immunohistochemical receptor expression corresponds to tracer uptake. Furthermore, the negative correlation between TV and IRS supports the hypothesis that SSTR subtypes other than SSTR2A exert a predominant influence, consistent with findings in other tumor types [23, 24]. So TLU might reflect the tumors functional or endocrine behavior, but the role of SSTR2A in PMT remains unclear and needs further investigation. Conversely, the correlation of TLU and TmP/GFR would suggest that lower TmP/GFR is potentially associated with a higher chance of detecting PMT on SSTR-directed PET/CT, due to a larger size and more intense SSTR expression. We found no significant correlation between other PET derived parameters and laboratory markers. As this analysis was intended for hypothesis generation rather than confirmatory inference, no adjustment for multiple comparisons was applied. The findings should therefore be regarded as preliminary and interpreted accordingly. Consistently, Paquet et al. found no significant correlations between SUVmax and TV with phosphorus, calcium, FGF23 and PTH serum levels [25]. Concordant to Kato et al., we found no differences in FGF23 and calcium levels between patients with and without detectable PMT [12], which is most likely due to a high level of inter- and intraindividual variability of values depending on current treatment, i.e. (id est) dosing of phosphate and active Vitamin D and sampling time in relation to the last dose.

The association of TLU of PMT in SSTR-directed PET/CT with laboratory indicators of phosphate wasting might add diagnostic accuracy and guidance when to order the PET/CT scan. Our findings are in line with the comprehensive review by Minisola et al., the expert recommendations by Brandi et al. and Dahir et al. as well as the global guidance by de Beur et al., which emphasize the role of biochemical markers in TIO diagnosis [1, 2, 7, 26]. However, given the small sample sizes, most results provide initial evidence and validation in further studies is required.

### PMT vs Fracture

Our study demonstrated significant difference in SUVmax and SUVpeak values between PMTs and bone fractures in [^68^Ga]Ga-DOTATOC PET/CT. For generating new hypothesis, we determined a SUVmax threshold of 7.6 for distinguishing between PMT and fractures and the threshold shows both a high sensitivity and specificity. This could be of clinical utility in reducing false-positive findings and increasing diagnostic accuracy, considering the fact that patients frequently show tracer uptake in fractures besides PMT [27]. Kato et al. reported that it could be difficult to differentiate between true PMT and fractures [12]. Parghane and Basu reported a case of false-positive osseous findings in [^68^Ga]Ga-DOTATATE PET/CT with SUVmax values of 5.42, 3.84 and 2.3, respectively [28]. Based on our proposed SUVmax threshold of 7.6, these lesions could have been identified as non-PMT tracer accumulation. However, this cutoff value is derived from a limited cohort, and validation in a larger study is warranted.

### Follow Up

Improvement of clinical symptoms was an early and reliable marker for successful therapy. Follow-up of patients after surgical resection showed a marked improvement in phosphate levels, confirming the effectiveness of surgical intervention which is inevitably dependent on the success of functional imaging. For those with biochemically confirmed TIO without detectable PMT, directly blocking FGF23 with burosumab provides a sound and well-tolerated therapeutic option for managing these patients. However, we also suggest to re-evaluate the correct execution of the PET/CT, especially with regard to the use of a SSTR-directed tracer and the complete coverage particularly of the peripheral limbs. In case of insufficient imaging and in line with consented recommendations, we encourage a follow-up imaging after one year to identify previously missed PMT. Even though we would not expect this imaging to be obscured by concomitant treatment, data in that regard is lacking.

### Limitations

Despite the promising results, our study has several limitations. Given the retrospective study design, CT acquisition protocols were not uniform, leading to minor heterogeneity in imaging analysis. Tumor volume was delineated using a 40% isocontour threshold. This method was applied given its established reproducibility; however, partial volume effects may lead to SUV underestimation in small or heterogeneous lesions, which should be considered when interpreting the volumetric data. Nerver the less, the threshold was chosen to provide a practical method that can be easily adopted in clinical practice. Regarding FGF23, assay heterogeneity limits comparability of absolute values. Although relative deviations from the upper limit of normal were calculated to partially address this limitation, inter-assay variability may have obscured a potential associations between FGF23 and PET derived parameters. The retrospective nature and relatively small sample size limit the generalizability of the findings. However, these limitations are common in TIO research due to the rarity of the condition. Future studies ideally with larger, prospective cohorts should re-evaluate our observed correlations and the suggested SUVmax threshold and implement standardized follow-up protocols in case of negative findings.

## Conclusion

In conclusion, this study emphasizes the value of SSTR-directed PET/CT with [^68^Ga]Ga-DOTATOC in the diagnosis and treatment planning of TIO, supporting its role as a first-line imaging modality. The findings offer new insights into the relationship between imaging and biochemical parameters. TLU should be documented during interpretation of SSTR-directed PET/CT and a threshold of 7.6 for SUVmax may be considered in case of ambiguous findings in bones. Beside phosphorus levels, the renal parameters TRP and TmP/GFR are suitable parameters for follow-up. The integration of PET derived TLU, along with the renal parameters TmP/GFR and TRP could improve diagnosis and treatment planning in this challenging condition.

## Supplementary Information

Below is the link to the electronic supplementary material.

ESM 1 DOCX (133 KB)

## Data Availability

All research data are available from the corresponding author upon reasonable request.
