# Supplementary material for: Diagnostic Performance of Somatostatin Receptor-directed PET/CT for Tumor-induced Osteomalacia
Source: Mol Imaging Biol. 2026 May 4;28(3):513–22. doi: 10.1007/s11307-026-02101-z (PMC13337602; doi:10.1007/s11307-026-02101-z)
Supplement: Supplementary file 1 — DOCX (133 KB) [file 11307_2026_2101_MOESM1_ESM.docx]

# *Supplemental Material*

Supplemental Table 1 patient based biochemical and PET-derived values­­­

| Patient | Ca [mmol/l] | Ph [mmol/l] | FGF23 [%] | NTx [BCE/l] | TRP  [%] | TmP/GFR [mmol/l] | SUVmax PMT | SUVpeak PMT | SUVmean PMT | TV [ml] PMT | TLU [ml] PMT | SUVmax  fracture | SUVpeak fracture |
| --- | --- | --- | --- | --- | --- | --- | --- | --- | --- | --- | --- | --- | --- |
| 01 | 2.5 | 0.66 | 329 | 33.5 | 74 | 0.66 | 15.79 | 7.14 | 8.88 | 1.19 | 10.61 | 1.62 | 1.01 |
| 02 | 2.69 | 1.41 | 800 | 14.3 | 90 | 1.4 | 0.00 | 0.00 | 0.00 | 0.00 | 0.00 | 0.00 | 0.00 |
| 03 | 2.35 | 1.06 | 452 | 14.2 | 58 | 1.06 | 60.28 | 17.27 | 38.35 | 0.25 | 9.54 | 2.55 | 1.66 |
| 04 | 2.32 | 0.57 | 299 | 25.0 |  |  | 7.08 | 4.42 | 4.27 | 1.74 | 7.43 | 4.2 | 2.39 |
| 05 | 2.32 | 0.69 | 207 | 13.2 | 86 | 0.69 | 9.11 | 5.09 | 5.47 | 1.37 | 7.49 | 7.24 | 4.76 |
| 06 | 2,32 | 0.53 | 1214 | 21.5 | 42 | 0.53 | 33.36 | 15.75 | 18.82 | 2.44 | 45.9 | 2.73 | 1.23 |
| 07 | 2.18 | 0.41 | 85 | 13.2 | 61 | 0.41 | 75.15 | 39.56 | 48.29 | 2.04 | 98.53 | 5.76 | 2.9 |
| 08 | 2.35 | 0.53 | 102 | 23.0 | 61 | 0.53 | 14.8 | 7.41 | 8.32 | 1.34 | 11.18 | 3.8 | 1.54 |
| 09 | 2.37 | 0.52 | 146 | 12.7 |  |  | 7.82 | 4.27 | 4.82 | 1.74 | 8.39 | 2.85 | 1.88 |
| 10 | 2.54 | 0.87 | 1818 | 39.0 | 82 | 0.87 | 0.00 | 0.00 | 0.00 | 0.00 | 0.00 | 0.00 | 0.00 |
| 11 | 254 | 0.67 | 182 | 22.1 | 59 | 0.67 | 9.61 | 8.83 | 5.28 | 2.44 | 12.87 | 4.56 | 2.69 |
| 12 | 2.47 | 0.55 | 340 | 29.8 | 80 | 0.55 | 92.36 | 42.7 | 59.4 | 1.41 | 83.76 | 5.1 | 3.34 |
| 13 | 2.23 | 1.07 | 1429 | 7.0 |  | 1.07 | 0.00 | 0.00 | 0.00 | 0.00 | 0.00 | 0.00 | 0.00 |
| 14 | 2.25 | 0.60 | 44 | 22.4 | 62 | 0.60 | 5.5 | 2.72 | 3.11 | 1.2 | 3.75 | 2.84 | 1.73 |
| 15 | 2.57 | 0.47 | 490 | 20.3 | 86 | 0.47 | 36.77 | 20.34 | 20.86 | 1.84 | 38.4 | 7.35 | 4.28 |
| 16 | 2.39 | 0.55 | 100 |  | 87 | 0.55 | 0.00 | 0.00 | 0.00 | 0.00 | 0.00 | 0.00 | 0.00 |
| 17 | 2.35 | 0.49 | 77 |  | 83 | 0.49 | 0.00 | 0.00 | 0.00 | 0.00 | 0.00 | 0.00 | 0.00 |
| 18 | 2.27 | 0.80 | 150 |  | 87 | 0.80 | 0.00 | 0.00 | 0.00 | 0.00 | 0.00 | 0.00 | 0.00 |
| 19 | 2.54 | 0.89 | 118 | 16.4 | 89 | 0.89 | 0.00 | 0.00 | 0.00 | 0.00 | 0.00 | 0.00 | 0.00 |
| 20 | 2.29 | 0.77 | 90 | 6.7 | 74 |  | 0.00 | 0.00 | 0.00 | 0.00 | 0.00 | 0.00 | 0.00 |

Abbreviations: Ca: calcium; Ph: phosphate; FGF23: fibroblast growth factor 23; NTx: N-terminal telopeptide; TRP: tubular reabsorption of phosphate; TmP/GFR: tubular maximum phosphate reabsorption capacity; SUV: standardized uptake value; TV: tumor volume; TLU: Total lesion uptake

Supplemental Table 2 PET parameter and laboratory values

| PET Parameter (n = 12) | | median | range |
| --- | --- | --- | --- |
| PMT | SUVmax | 15.30 | 5.50 – 92.36 |
|  | SUVpeak | 8.12 | 2.72 – 42.70 |
|  | SUVmean | 8.6 | 3.11 – 59.40 |
|  | TV [ml] | 1.58 | 0.25 – 2.44 |
|  | TLU [ml] | 10.90 | 3.75 – 98.53 |
| Hottest fracture | SUVmax | 4.00 | 1.62 – 7.35 |
|  | SUVpeak | 2.14 | 1.01 – 4.76 |
| Laboratory values (n = 20) | Normal range | median | range |
| Calcium [mmol/l] | 2.1 – 2.6 | 2.36 | 2.18 – 2.57 |
| Phosphorus [mmol/l] | 0.87 -1.45 | 0.63 | 0.41 -1.06 |
| ALK [U/l] | 53.00 – 128.00 | 205.50 | 67.00 – 481.00 |
| PTH [pg/ml] | 15.00 – 57.00 | 51.80 | 12.90 – 284.50 |
| Vitamin D [ng/ml] | 20.00 – 40.00 | 27.00 | 11.00 – 54.00 |
| FGF23 [%] | 35 – 100 | 176 | 44 – 1214 |
| N-Telopeptide [BCE/l] | 5.40 – 24.20 | 20.90 | 12.70 – 33.50 |
| TRP [%] | 82 – 90 | 74 | 42 – 86 |
| TmP/GFR [mmol/l] | 0.80 – 1.60 | 0.66 | 0.41 – 1.10 |

Abbreviations: ALK: alkaline phosphatase; FGF23: fibroblast growth factor 23; PMT: phosphaturic mesenchymal tumors; PTH: parathyroid hormone; SUV: standardized uptake value; TLU: total lesion uptake; TmP: tubular maximum of phosphate reabsorption; TmP/GFR: tubular maximum of phosphate reabsorption capacity; TRP: tubular reabsorption of phosphate; TV: tumor volume

Supplemental Table 3 PMT in SSTR – directed PET/CT

|  | | number [n] | percentage [%] |
| --- | --- | --- | --- |
| Detection of PMT in SSTR-directed PET/CT | | 12 | 60 |
| PMT positive patients (n = 12) | | number [n] | percentage [%] or range |
| Localization of PMT in SSTR- directed PET/CT | lower limb | 6 | 50 |
|  | trunk | 4 | 33 |
|  | pelvis | 1 | 8 |
|  | hand/foot | 1 | 8 |
| Origin of PMT | bone | 4 | 19 |
|  | soft tissue | 8 | 38 |
| Pathological fractures parallel to detected PMT | | 23 | 3 - 69 |

Abbreviations: PMT: phosphaturic mesenchymal tumors; SSTR: somatostatin receptor

Supplemental Table 4: IRS for all PMT+ patients (n = 12). Proportion: 0 for 0 %, 1 for 1-9 %, 2 for 10-50 %, 3 for 51 – 80 % and 4 ≥ 80 %. Intensity: 1 for weak intensity, 2 for medium intensity and 3 for strong intensity.

| Patient | Proportion [%] | intensity | IRS [proportion x intensity] |
| --- | --- | --- | --- |
| 01 | 4 | 2 | 8 |
| 03 | 4 | 3 | 12 |
| 04 | n.e. | n.e. | n.e. |
| 05 | 3 | 1 | 3 |
| 06 | 3 | 2 | 6 |
| 07 | 2 | 2 | 4 |
| 08 | 4 | 3 | 12 |
| 09 | 2 | 3 | 6 |
| 11 | 1 | 1 | 1 |
| 12 | 3 | 3 | 9 |
| 14 | 2 | 1 | 2 |
| 15 | 3 | 2 | 6 |

Abbreviations: IRS: immunoreactive score; n.e.: not evaluable; PMT: phosphaturic mesenchymal tumors; SSTR: somatostatin receptor

Supplemental Table 5: Spearman rank test PET values and biochemical values

| Correlation parameters | | number | r-value | p-value |
| --- | --- | --- | --- | --- |
| SUVmax | Calcium [mmol/l] | 12 | 0.22 | 0.49 |
|  | Phosphorus [mmol/l] | 12 | -0.25 | 0.44 |
|  | FGF23 [%] | 12 | 0.48 | 0.12 |
|  | N-Telopeptide [BCE/l] | 12 | -0.01 | 1.00 |
|  | TRP [%] | 10 | -0.08 | 0.83 |
|  | TmP/GFR [mmol/l] | 10 | -0.38 | 0.27 |
| SUVpeak | Calcium [mmol/l] | 12 | 0.24 | 0.44 |
|  | Phosphorus [mmol/l] | 12 | 0.27 | 0.39 |
|  | FGF23 [%] | 12 | 0.46 | 0.13 |
|  | N-Telopeptide [BCE/l] | 12 | -0.01 | 1.00 |
|  | TRP [%] | 10 | -0.09 | 0.82 |
|  | TmP/GFR [mmol/l] | 10 | -0.46 | 0.19 |
| SUVmean | Calcium [mmol/l] | 12 | 0.17 | 0.60 |
|  | Phosphorus [mmol/l] | 12 | -0.24 | 0.45 |
|  | FGF23 [%] | 12 | 0.48 | 0.12 |
|  | N-Telopeptide [BCE/l] | 12 | -0.04 | 0.92 |
|  | TRP [%] | 10 | 0.01 | 1.00 |
|  | TmP/GFR [mmol/l] | 10 | -0.37 | 0.29 |
| TV [ml] | Calcium [mmol/l] | 12 | 0.04 | 0.90 |
|  | Phosphorus [mmol/l] | 12 | -0.05 | 0.08 |
|  | FGF23 [%] | 12 | 0.11 | 0.74 |
|  | N-Telopeptide [BCE/l] | 12 | -0.27 | 0.39 |
|  | TRP [%] | 10 | -0.18 | 0.62 |
|  | TmP/GFR [mmol/l] | 10 | -0.49 | 0.15 |
| TLU [ml] | Calcium [mmol/l] | 12 | 0.23 | 0.48 |
|  | Phosphorus [mmol/l] | 12 | -0.51 | 0.09 |
|  | FGF23 [%] | 12 | 0.30 | 0.34 |
|  | N-Telopeptide [BCE/l] | 12 | -0.01 | 1.00 |
|  | TRP [%] | 10 | -0.14 | 0.70 |
|  | TmP/GFR [mmol/l] | 10 | -0.71 | 0.03 |

Abbreviations: FGF23: fibroblast growth factor 23; SUV: standardized uptake value; TLU: total lesion uptake; TmP/GFR: tubular maximum of phosphate reabsorption capacity; TRP: tubular reabsorption of phosphate; TV: tumor volume


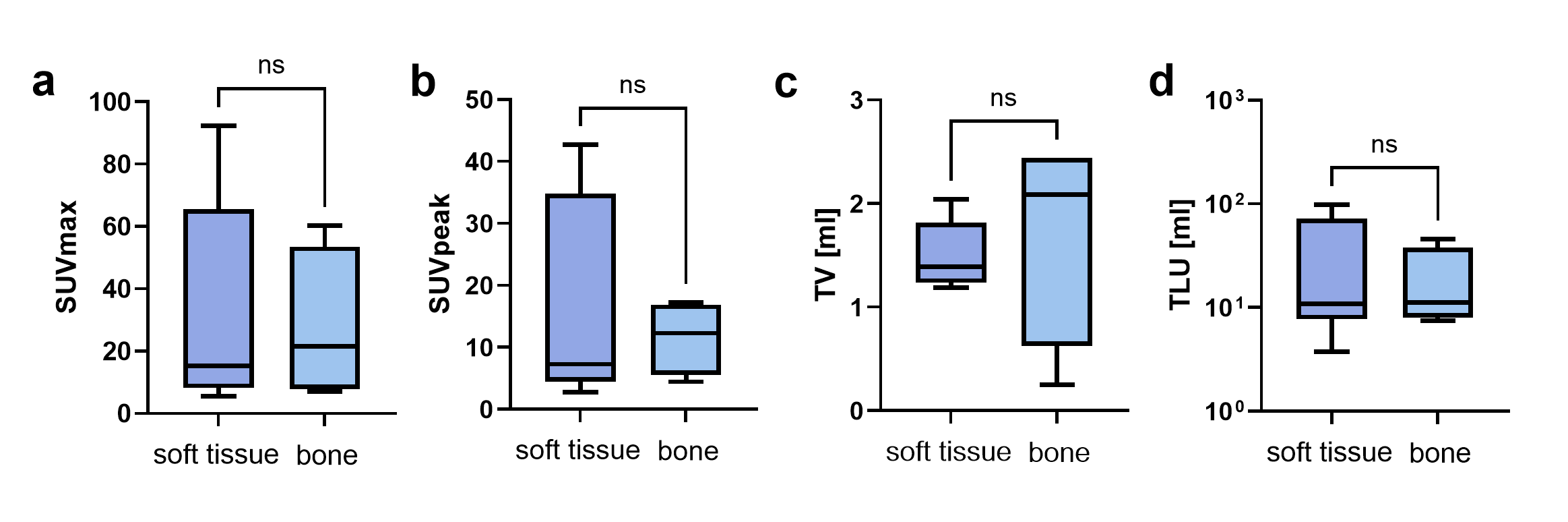


**Supplemental Fig. 1** No significant differences in PMT of osseous and soft tissue origin in PET-derived parameters SUVmax (A), SUVpeak (B), TV (C) and TLU (D).

Abbreviations: PMT: phosphaturic mesenchymal tumors; SUV: standardized uptake value; TLU: total lesion uptake; TV: tumor volume


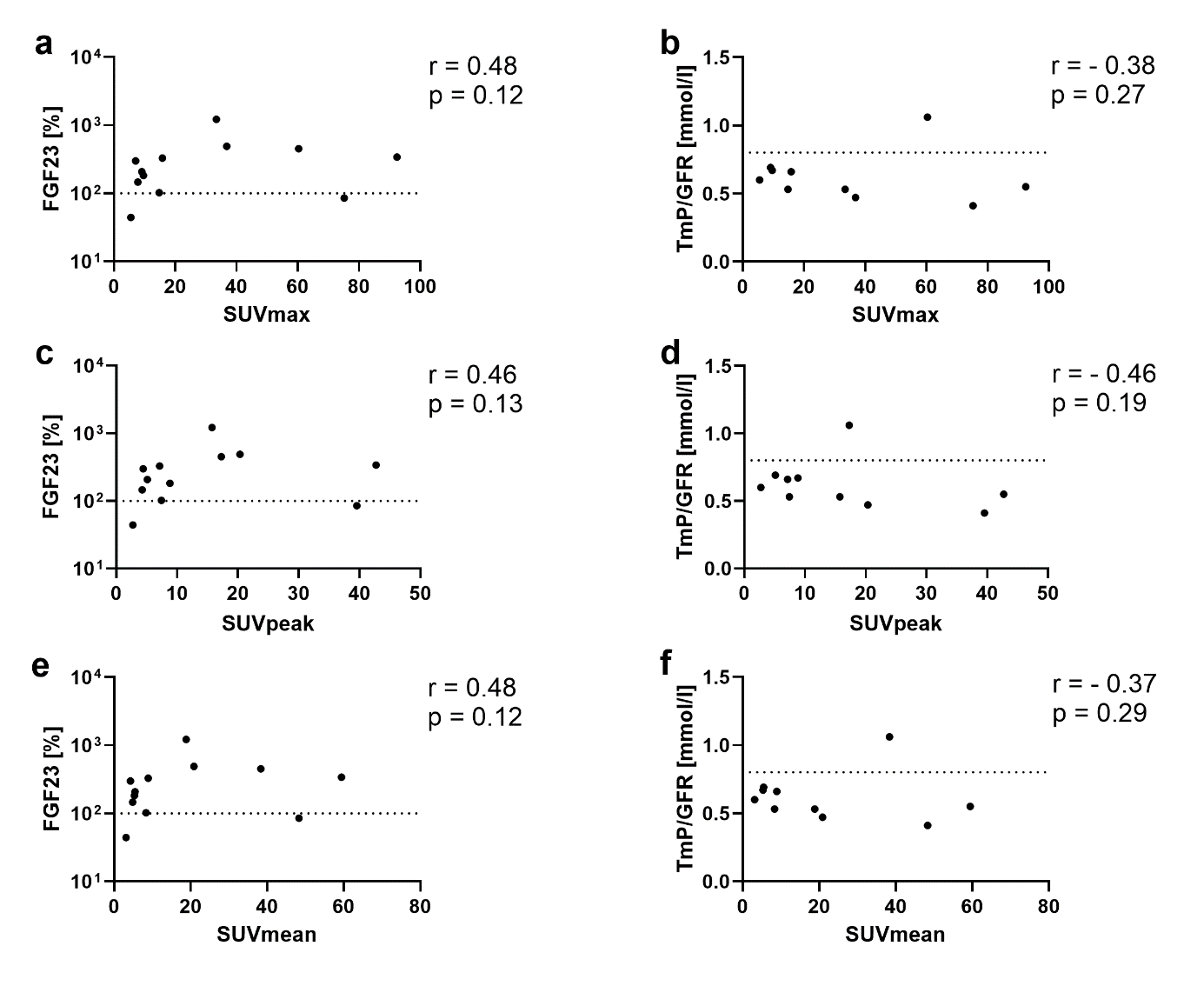


**Supplemental Fig. 2** Correlation of PET derived values SUVmax (A-B), SUVpeak (C-D), SUVmean (E-F) and biochemical values FGF23 (A; C; E) and TmP/GFR (B; D; F).

Abbreviations: FGF23: fibroblast growth factor 23; SUV: standardized uptake value; TmP/GFR: tubular maximum of phosphate reabsorption capacity
